# Diagnostic Accuracy of Ultrasound Imaging and Shear Wave Elastography to Discriminate Patients with Chronic Neck Pain from Asymptomatic Individuals

**DOI:** 10.3390/healthcare12191987

**Published:** 2024-10-05

**Authors:** Gustavo Plaza-Manzano, César Fernández-de-las-Peñas, María José Díaz-Arribas, Marcos José Navarro-Santana, Sandra Sánchez-Jorge, Carlos Romero-Morales, Juan Antonio Valera-Calero

**Affiliations:** 1 Department of Radiology, Rehabilitation and Physiotherapy, Faculty of Nursery, Physiotherapy and Podiatry, Universidad Complutense de Madrid, 28040 Madrid, Spain; gusplaza@ucm.es (G.P.-M.); mjdiazar@med.ucm.es (M.J.D.-A.); marconav@ucm.es (M.J.N.-S.); juavaler@ucm.es (J.A.V.-C.); 2 Grupo InPhysio, Instituto de Investigación Sanitaria del Hospital Clínico San Carlos (IdISSC), 28040 Madrid, Spain; 3 Department of Physical Therapy, Occupational Therapy, Rehabilitation and Physical Medicine, Universidad Rey Juan Carlos, 28922 Alcorcón, Spain; 4 Cátedra en Docencia, Clínica e Investigación en Fisioterapia: Terapia Manual, Punción Seca y Ejercicio Terapéutico, Universidad Rey Juan Carlos, 28922 Alcorcón, Spain; 5 Faculty Health Sciences, Universidad Francisco de Vitoria, 28223 Madrid, Spain; s.sjorge.prof@ufv.es; 6 Faculty of Sport Sciences, Universidad Europea de Madrid, Villaviciosa de Odón, 28670 Madrid, Spain; carlos.romero@universidadeuropea.es

**Keywords:** sensitivity and specificity, neck pain, elasticity imaging techniques, ultrasonography

## Abstract

Objectives: The aim of this study was to determine and compare the capability of several B-mode ultrasound (US) and shear wave elastography (SWE) metrics to differentiate subjects with chronic non-specific neck pain from asymptomatic subjects. Methods: A diagnostic accuracy study recruiting a sample of patients with chronic neck pain and asymptomatic controls was conducted. Data collection included sociodemographic information (i.e., gender, age, height, weight and body mass index), clinical information (pain intensity assessed using the Visual Analogue Scale and pain-related disability using the Neck Disability Index) and B-mode ultrasound and shear wave elastography features of the cervical multifidus muscle (cross-sectional area, perimeter, mean echo intensity, fat infiltration, shear wave speed and Young’s modulus). After analyzing between-group differences for left/right sides, cases and controls, and males and females, the area under the receiver operating characteristic (ROC) curve, the optimal cut-off point, the sensitivity, the specificity, the positive likelihood ratio (LR) and negative LR for each metric were calculated. A total of 316 individuals were recruited in this study (*n* = 174 cases with neck pain and *n* = 142 asymptomatic controls). Results: No significant differences (*p* > 0.05) were found between cases and controls for most variables, except for fatty infiltration, which was significantly higher in chronic neck pain cases (*p* < 0.001). Gender differences were significant across all US and SWE metrics (all, *p* < 0.001 except *p* = 0.015 for fatty infiltrates). A slight asymmetry was observed between the left and right sides for area (*p* = 0.038). No significant interactions between group, gender and side (all metrics, *p* > 0.008) were identified. Fatty infiltration was the most effective discriminator, with a ROC value of 0.723, indicating acceptable discrimination. The optimal cut-off point for fatty infiltration was 25.77, with a moderate balance between sensitivity (59.8%) and specificity (20.5%). However, its positive likelihood ratio (LR) of 0.75 suggests limited usefulness in confirming the condition. Conclusions: Fatty infiltration was significantly higher in individuals with chronic idiopathic neck pain compared to those without symptoms, while other muscle metrics were similar between both groups. However, since fat infiltration had moderate diagnostic accuracy and the other metrics showed poor discriminatory power, US cannot be used solely to discriminate patients with idiopathic neck pain.

## 1. Introduction

Neck pain is a prevalent disorder worldwide, affecting up to 3551.1 out of 100,000 people in 2017 [1] and refers to any discomfort, tenderness or pain in the neck region [2]. Since there is an increasing prevalence in neck pain over time, there is a growing need to investigate this condition further [3]. Despite the generally favorable natural course of neck pain, its tendency for recurrence and chronicity poses significant challenges [4]. This condition imposes a substantial economic and social burden, with combined healthcare expenditures for lower back and neck pain in the United States reaching approximately USD 134.5 billion in 2016 [3]. Moreover, neck pain leads to significant work absenteeism, affecting millions of individuals and thereby impacting productivity and economic stability [5].

The complexity of neck pain is evident in its diverse causes, ranging from acute traumatic injuries like whiplash to chronic issues such as degenerative disc disease or arthritis [6,7,8]. Psychological factors, including stress and depression, as well as physical factors like poor posture and prolonged sedentary behavior, further complicate its diagnosis and treatment [9]. Although clinical guidelines exist for the appropriate use of imaging studies in patients with neck pain, differentiating between acute and chronic stages, and between traumatic and non-traumatic patients [10,11], these studies often fail to correlate structural findings with those symptoms reported by patients [12].

The morphology and histology of the cervical multifidus (CM) have been explored previously in several studies that have found significant differences between individuals with neck pain and asymptomatic subjects, as well as an association with neck pain clinical severity indicators [13,14,15,16], in multiple conditions including whiplash-associated disorders [17], chronic idiopathic neck pain [18], cervical radiculopathy [19], cervical spondylosis [20] and spondylotic myelopathy [21,22]. Considering that its primary functions are cervical spine extension and stabilization [23], the morphological and histological changes in the CM are suggested to cause impaired sensorimotor function, poor postural stability, and increased disability [24,25]. Scientific evidence strongly advocates for a comprehensive analysis of these metrics during the initial evaluation of patients with neck pain [12]. Such an assessment could not only provide a baseline understanding of the patient’s condition, but also aids in tailoring therapeutic interventions focusing on therapeutic exercise programs. Furthermore, periodic reassessments during consecutive follow-ups are crucial to determine the efficacy of the treatment modalities employed and to make necessary adjustments [26]. In fact, a previous study focusing on specific neck muscles stiffness [27] found that individuals with chronic neck pain exhibited greater stiffness compared to healthy controls (although these differences are controversial for the CM muscle [23,28]) and concluded that this metric is crucial as it may reflect changes in neuromuscular control and muscle material properties. These differences in muscle morphology, histology and stiffness emphasizes the importance of evaluating US metrics in diagnosing and designing rehabilitation programs for neck pain sufferers and may lead to more tailored and effective treatment strategies [23].

Since there is no available evidence analysing the diagnostic utility of examining the CM muscle with US or SWE, the objective of this study was to determine the capability of B-mode US and SWE to differentiate individuals with chronic non-specific neck pain by analyzing the area under the receiver operating characteristic (ROC) curve, the optimal cut-off point, the sensitivity, the specificity, the positive likelihood ratio (LR) and negative LR for each metric.

## 2. Methods

### 2.1. Study Design

A cross-sectional observational study, with a diagnostic accuracy design, to detect differences in CM muscle morphology, histology and stiffness between individuals with chronic idiopathic neck pain and asymptomatic controls was conducted. To ensure an appropriate level of quality was maintained, this study adhered to the Updated List of Essential Items for Reporting Diagnostic Accuracy Studies (STARD 2015) [29] and the Enhancing the QUAlity and Transparency Of health Research (EQUATOR) guidelines [30]. In addition, the study protocol and the ethical considerations including the participants’ rights were supervised and approved by the Local Ethics Committee of a third-party university prior to starting the data collection.

### 2.2. Participants

Two groups of participants were defined: one of asymptomatic individuals (controls) and another of individuals suffering from bilateral chronic idiopathic neck pain (cases). The recruitment was conducted across various universities located in Madrid (Spain) by posting announcements between January 2024 and June 2024. By using a QR code, potential volunteers were informed about how to contact the research team and the requirements to participate, and were also provided with the fully informed consent document containing all the study information.

Common eligibility criteria for both cohorts included being aged between 18 and 65 years old, absence of previous history of traumatic events (e.g., whiplash, fractures or fissures), surgeries or neuropathic disorders of the head/spine, and not being under any treatment potentially affecting the muscle tone or psychological disorders (e.g., physiotherapy, or drugs such as muscle relaxants, anxiolytics, or antidepressants). To be allocated to the control group, participants had to confirm no episodes of neck pain for at least the previous year. On the other hand, participants allocated to the cases group had to report chronic (in accordance with recognized guidelines for the classification of neck pain [12], participants had to report symptoms for at least 12 weeks of duration) and bilateral pain (as previous studies reported that bilateral pain is associated with higher levels of sensitization and poorer functionality compared with individuals with unilateral pain) [31,32], a minimum pain intensity of 3.5 points on the Numerical Pain Rate Scale (as this is the cut-off for determining at least moderate pain intensity [33]) and 15 points on the Neck Disability Index (which is the cut-off point for disability sensitivity and specificity [34]).

### 2.3. Sample Size Estimation

For the sample size estimation, the G*Power software v.3.1 was utilized. The two-tailed *t*-test a priori analysis for calculating mean differences between two independent samples resulted in a total sample of *n* = 128 participants (*n* = 64 per group) setting a standard effect size of d = 0.5, α = 0.05, β = 0.8 and an allocation ratio of N2/N1 = 1.

### 2.4. Outcomes

#### 2.4.1. Demographic and Clinical Data

All participants filled out a standardized document for collecting demographic and clinical data. Participants were asked about their age (years), *height* (m) and *weight* (kg). Later, body mass index was calculated for analyses (BMI = $\frac{weight}{{height}^{2}}$ kg/m^2^) [35].

Pain-related disability and pain intensity were assessed using the Neck Disability Index (NDI) and the Numeric Pain Rating Scale (NPRS), respectively. The NDI is a self-report questionnaire, adapted to multiple languages, consisting of 10 items evaluating how neck pain interferes with daily living physical tasks and related complaints (i.e., headaches or concentration impairments). Final scores range from 0 to 100 and can be used to classify the disability as “mild” (10–28 points), “moderate” (30–48 points), “severe” (50–68 points) or complete (>70 points) [36]. The NPRS is a pain intensity scale where all the participants in the cases group were asked to rate their pain intensity from 0 (no pain) to 10 (the worst pain imaginable). To improve the accuracy of the measurements, a mean average of 3 different moments (the current pain intensity during the data collection, and the highest and lowest pain intensities perceived during the previous week) was calculated [37]. This scale can be used to classify pain intensity as “mild” (≤5 points), “moderate” (6–7 points) or “severe” (≥8 points) [38].

#### 2.4.2. Cervical Multifidus Ultrasound Imaging Acquisition

The CM muscle arises from the superior articular processes of the C4 to C7 vertebrae, inserts into the spinous processes two to four segments higher than its origin and spans over the lamina of the vertebrae beneath it [39]. The B-mode and SWE protocols used to examine the CM muscle at the C4–C5 level were previously tested and demonstrated acceptable reliability [23,40]. Images were collected bilaterally using a US device, Logiq E9 with a linear transducer (6–15 MHz ML-6-15-D) by a single examiner (+10 years of experience using US and +5 years using SWE for musculoskeletal assessments). Standard console settings, including a frequency of 12 MHz, gain of 65 dB and depth of 4.5 cm, were used for all acquisitions.

Participants were relaxed in the prone position (to avoid muscle contraction and reduce stiffness variability) with a neutral cranio-cervical angle, a pillow under their ankles and the upper limb resting at 90° of shoulder abduction and elbow flexion. The examiner commenced with the manual identification of the C2 spinous process. The transducer was then positioned horizontally to achieve a B-mode short-axis image beginning at C2. The transducer was gradually moved downward until the C4 vertebra came into view. Following this, a lateral movement was performed to center on the C4 over the articular pillar. The imaging aimed to capture the CM muscle at the juncture where the most superficial part of the spinous tubercle’s cortical surface and the apex of the C4/C5 joint were both visible. It was crucial to ensure that the muscle appeared perpendicular at the center of the image (a pivotal condition to avoid an anisotropic artifact), applying minimal pressure to acquire a clear image.

#### 2.4.3. B-Mode Ultrasound and Shear Wave Elastography Analysis

**Figure 1:**
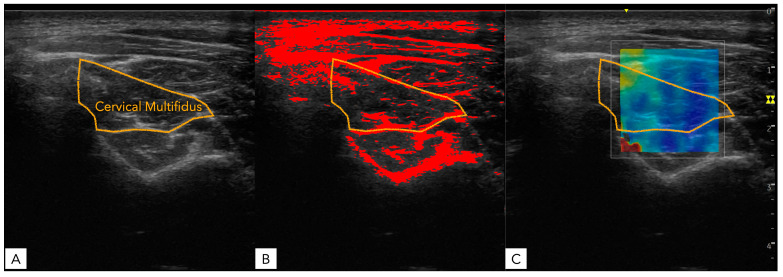
Ultrasound imaging of the cervical multifidus muscle acquired at the C4–C5 level: (**A**) raw image; (**B**) fat infiltration calculation; (**C**) shear wave elastography.

All images were transferred in DICOM format to ImageJ offline software v.1.42 (National Institutes of Health, Bethesda, MD, USA) and converted from RGB to 32-bit (256 grayscale) format to measure muscle morphology and brightness. First, the CM muscle was outlined within the spinous process of the C4, between the medial internal fascia, short rotators, and semispinalis at the bottom and superomedial boundary, respectively (Figure 1A). Next, a brightness range was selected to determine the upper cut-off echo intensity for isolating fatty infiltration (and the normal connective tissue within the muscle), using the subcutaneous tissue as a reference for each image. Finally, the muscle cross-sectional area (CSA), perimeter, mean echo intensity and percentage of fatty infiltration were automatically calculated and recorded (Figure 1B). For SWE analysis, the same contouring procedure was followed using the US equip software to obtain shear wave speed (SWS) and Young’s modulus (Figure 1C).

### 2.5. Statistical Analysis

Data analyses were performed using the Statistical Package for the Social Sciences (SPSS) version 27.1 for Mac OS (Armonk, NY, USA), setting the two-tailed significance level at *p* < 0.05 for all the analyses.

The mean of the ultrasound metrics was calculated using both sides and descriptive statistics were used for each group (means and standard deviations). Between-groups differences were calculated and analyzed using a multivariate general lineal model with analysis of covariance (ANCOVA), including the group, side and gender as fixed factors. *p* values were assumed to be significant at <0.05 for individual fixed factors. For analyses involving the interaction between gender, group and side, a Bonferroni correction was applied to account for multiple comparisons. Since six comparisons were made, the significance level was adjusted to *p* < 0.008 (0.05/6) to control for type-I errors and ensure the overall chance of incorrectly finding a significant result remained below 0.05.

Finally, the capability of ultrasound metrics to differentiate cases with neck pain from asymptomatic subjects was assessed by analyzing the area under the receiver operating characteristic (ROC) curve, considering a ROC curve value of ≥0.7 as acceptable discrimination [41]. The optimal cut-off point for each measure ratio was identified using the Youden index. The reported metrics included sensitivity, specificity, positive likelihood ratio (LR) and negative LR. The validity was deemed acceptable if a sensitivity of at least 70% and a specificity of at least 50% were achieved [42].

## 3. Results

During the recruitment period, 316 individuals showed interest in participating in this study. Since no data were missed and no participants were excluded from this study, all participants were successfully analyzed (cases with neck pain: *n* = 174, 63.2% female; asymptomatic individuals: *n* = 142, 35.9% female). Table 1 presents the demographic and clinical features of the study participants, comparing differences between males and females within the cases and controls. Age was comparable between males and females in both cases (*p* = 0.957) and controls (*p* = 0.751), with no significant between-group difference (*p* = 0.214). Males were significantly taller than females in both groups (both, *p* < 0.001), and this difference was also significant between groups due to their gender distribution (*p* < 0.001). Although males weighed more than females in both groups (both, *p* < 0.001), the between-groups difference in weight was not significant (*p* = 0.133). BMI differences were significant between males and females in both cases (*p* = 0.008) and controls (*p* = 0.022), but not between groups (*p* = 0.600). Clinical severity descriptors within the cases group, such as NDI and VAS scores, showed no significant gender differences (NDI, *p* = 0.878; VAS, *p* = 0.052).

The sonographic features of the CM muscle, including area, perimeter, mean echo intensity, fatty infiltration, Young’s modulus and SWS, are summarized in Table 2. No significant between-groups differences were observed for area, perimeter, mean echo intensity, Young’s modulus or SWS (all, *p* > 0.05). However, fatty infiltration was significantly higher within cases with neck pain compared to the controls (*p* < 0.001). Regarding gender differences, significant differences were observed for all sonographic variables, with males generally showing larger values: area (*p* < 0.001), perimeter (*p* < 0.001), mean echo intensity (*p* < 0.001), fatty infiltration (*p* = 0.015), Young’s modulus (*p* < 0.001) and SWS (*p* < 0.001). The comparison between the left and right sides showed a significant difference only for area (*p* = 0.038), indicating a slight asymmetry. No significant differences were observed for the other variables: perimeter (*p* = 0.848), mean echo intensity (*p* = 0.259), fatty infiltration (*p* = 0.328), Young’s modulus (*p* = 0.912) and SWS (*p* = 0.699). The interaction analysis (group*gender*side) did not reveal any significant interactions for any of the variables, indicating that the observed effects of group, gender and side are independent of each other (all, *p* > 0.05).

Table 3 outlines the discriminant capacity of various ultrasound metrics in differentiating cases with chronic neck pain from asymptomatic individuals. The ROC analysis revealed that fatty infiltration had the highest discriminatory ability, with a ROC value of 0.723, indicating acceptable discrimination. The optimal cut-off point for fatty infiltration was 25.77, with a Youden Index of 0.392, suggesting a moderate balance between sensitivity (59.8%) and specificity (20.5%). The positive likelihood ratio (LR) was 0.75, indicating limited usefulness when the test is positive, and the negative LR was 1.96, reflecting a moderate ability to exclude chronic neck pain when the test is negative.

The other variables—area, perimeter, mean echo intensity, Young’s modulus and SWS—demonstrated poor discriminatory capacity, as all had ROC values below 0.7. Mean echo intensity and SWS had borderline significance with ROC values of 0.587 and 0.591, respectively. The sensitivity and specificity for these variables were low, with positive LRs indicating weak diagnostic utility. Specifically, the positive LR for SWS was 2.11, suggesting a slightly higher likelihood of chronic neck pain when positive, while the negative LR of 0.31 indicates moderate exclusion capacity when the test is negative. Despite these findings, the overall poor ROC values suggest that these metrics should not be solely relied upon for diagnosing chronic neck pain.

**Figure 2:**
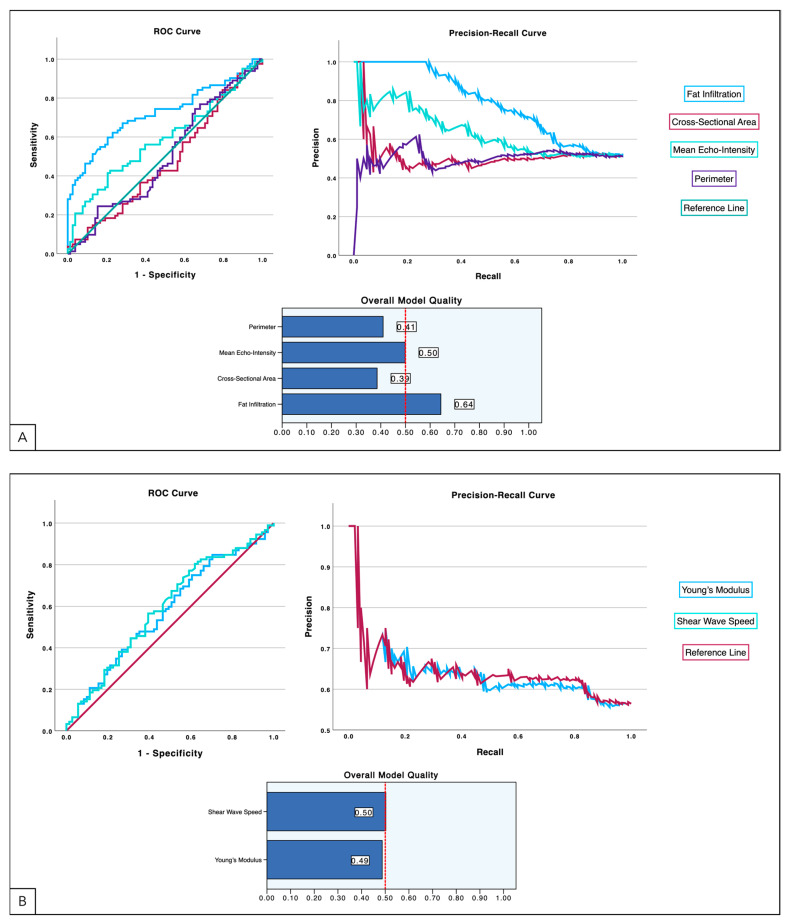
Comparison of model performance for B-mode ultrasound (**A**) and shear wave imaging (**B**) using ROC and Precision-Recall curves. The ROC curve shows the trade-off between sensitivity and specificity for each parameter, while the Precision-Recall curve further details the performances of these parameters. The bar charts quantify the overall model quality for each parameter.

To provide a more visual information, Figure 2 illustrates the discriminant capacity of CM B-mode ultrasound (Figure 2A) and shear wave elastography (Figure 2B) metrics by providing the ROC curves, Precision-Recall curves and a summary of the overall models’ quality. Fat infiltration stands out with the highest area under the curve (AUC), indicating superior discriminatory power compared to the other B-mode and SWE parameters. The Precision-Recall curves further highlight the predictive performance of these parameters, particularly in situations with imbalanced data. Fat infiltration maintains high precision across varying levels of recall, making it a reliable predictor. In contrast, muscle CSA and perimeter show a drop in precision as recall increases, indicating weaker performance. Thus, Young’s modulus and SWS, exhibit similar precision, but with a noticeable decline as recall grows, which might limit their utility in certain predictive models. The overall model quality, summarized in the bar charts, reinforces these observations. Fat infiltration emerges as the most robust parameter, achieving the highest score of 0.64. Meanwhile, SWS and Young’s modulus show nearly equivalent but moderate model quality scores of 0.50 and 0.49, respectively. This suggests that while they have some predictive power, they are less reliable than the muscle quality parameters, particularly fat infiltration.

## 4. Discussion

The most relevant findings of this study were that (1) cases exhibited a similar cross-sectional area, perimeter, MEI, YM and SWS; (2) fatty infiltration was significantly higher in cases with neck pain compared to the controls; (3) slight asymmetries were observed between the left and right sides for the area; and (4) fatty infiltration had the highest discriminative ability in distinguishing with moderate sensitivity and specificity between individuals with neck pain and those without (while other metrics such as area, perimeter, mean echo intensity, Young’s modulus and SWS had lower discriminatory capacity with ROC values below the acceptable cut-off point).

Although several anatomical structures can be sources of nociception in the neck (i.e., joints, bones, muscles, ligaments, neural structures and the intervertebral disc), only in a smaller subset of cases with neck pain can these be traced back to specific causes and require thorough clinical evaluation, as these can be indicative of more serious conditions. In most of neck pain sufferers, the tissue that is causing neck pain is unknown [12] and is classified as idiopathic neck pain or non-specific neck pain [43]. Consequently, imaging studies for cases with idiopathic neck pain often fail to identify any structural pathology related to their symptoms [44,45] and, due to the similar frequency of abnormal findings observed in cases and asymptomatic individuals and the lack of prognostic value, imaging modalities in the absence of neurologic deficits or other disease processes are not recommended [12]. Instead, clinicians must assess for the impaired function of muscles, connective tissues and nerves associated with identified pathological tissues when neck pain is present. Although subjective history, validated self-report questionnaires and physical examination tests provide valuable information, US provides information that is not accessible using manual palpation (muscle morphology and composition) [46] and SWE overcomes the subjectivity bias of physical examination to assess muscle stiffness [47].

Muscle morphology and composition are potential factors that may provide insight into mechanisms underlying idiopathic neck pain or its chronicity (as muscle structure has been linked to the onset and persistence of neck pain, greater disability, postural instability, poorer balance and worse functional recovery after surgical interventions) and could be a discriminator factor for differentiating asymptomatic individuals from chronic neck pain sufferers [46,48,49,50]. Recently, a case-control study [18] demonstrated that individuals with chronic idiopathic neck pain have greater muscle volume and fat infiltration (measured using magnetic resonance imaging) in their deep extensor muscles compared to age- and sex-matched controls. These differences were evident even when controlling for age and BMI (two factors that are known to influence fat infiltration in muscles [51,52]), and varied depending on the specific spinal level (with greater fat infiltration observed at the lowest cervical levels compared to higher levels [18]) and the fatty infiltration location within the segment (fat infiltration in the medial portion of the muscle is significantly larger in those with greater severity [53]). However, it should be noted that correlation does not imply causality, and fatty infiltration can be either the source of pain or a consequence of muscle inactivity and weakness due to pain.

Wang et al., [54] conducted a review discussing triggering factors contributing to fat infiltration. Aging is one of the primary contributors, as it leads to a reduction in muscle mass and strength and the development of myosteatosis. Metabolic (such as type-2 diabetes and obesity) and non-metabolic diseases (i.e., Duchenne muscular dystrophy, rheumatoid arthritis or viral infections) are closely related to higher levels of intramuscular fat. Other relevant factors include muscle injuries (as occur with whiplash-associated disorders, which impair the regeneration of muscle tissue), as well as muscle disuse, weakness and inactivity (commonly found in patients suffering chronic pain and leading to greater fat infiltration as a compensatory mechanism). This review also explored the cellular origins of fat infiltration in skeletal muscle, noting that both myogenic and non-myogenic cells contribute to this process. Fibro/adipogenic progenitors are identified as a major source of fat infiltration, as they can differentiate into fat cells during muscle damage or regeneration. Other cell types, such as endothelial cells, fibroblasts and pericytes, also contribute to fat formation under certain conditions. In terms of regulatory mechanisms, several molecular pathways are involved in controlling fat infiltration in muscle tissue, including the AMP-activated protein kinase pathway (regulating energy balance and inhibiting fat accumulation), the MAPK, Wnt/β-catenin and Hedgehog signaling pathways (regulating the differentiation of muscle progenitors and preventing excessive fat deposition) and microRNAs and long non-coding RNAs (regulators of fat deposition in skeletal muscle through their influence on gene expression).

In addition, while the overall muscle volume was higher in those with neck pain, the relative volume (muscle volume excluding fat infiltration) did not differ significantly between groups, suggesting that the increase in muscle volume observed in the neck pain group was primarily due to fat infiltration rather than an increase in lean muscle mass [18]. This variability highlights the need for comprehensive assessments that consider multiple muscles and spinal levels to accurately reflect the health of the cervical spine.

On the other hand, the relevance of muscle stiffness assessed with SWE is not clear. Dieterich et al. [28] measured muscle stiffness at five different sites including the CM in women with chronic non-specific neck pain and asymptomatic women. The comparisons revealed no significant differences in objective muscle stiffness between the two groups, despite the neck pain group reporting a greater subjective feeling of stiffness. In general, the most remarkable findings of our research were that most US and SWE metrics did not significantly differ between individuals with (cases) and without (controls) neck pain. The only exception was fatty infiltration, which was significantly higher within the neck pain group. This suggests that while most sonographic features are not effective in distinguishing between these groups, fatty infiltration may be a relevant indicator of chronic neck pain. Additionally, significant gender differences were observed across almost all sonographic variables, with males generally displaying larger area and perimeter, higher MEI, and greater fatty infiltration than females. Differences between the left and right sides of the muscle were minimal, with only the area showing a slight asymmetry.

After an extensive search in the relevant databases, we found that no published study has analyzed the diagnostic utility of examining the CM muscle, making this the first report to use US and SWE to identify muscle metrics that could accurately differentiate subjects with chronic non-specific neck pain from asymptomatic individuals. Although a previous review examined the diagnostic values associated with the subjective history and self-report items used in identifying common cervical conditions, it did not include idiopathic neck pain due to lack of diagnostic accuracy studies [55]; however, relevant neck pain conditions were analyzed and can be discussed to compare our results. The review found that cervical radiculopathy is generally supported by specific self-report symptoms such as shoulder/scapular pain and neck movement that improves symptoms, alongside physical examination findings like electromyography, which has a sensitivity and specificity greater than 70%. For degenerative joint disease, diagnostic accuracy largely hinges on symptoms like hand radiculopathy and hand numbness, which demonstrate high specificity and can effectively rule in the condition. In contrast, cervical myelopathy shows higher diagnostic accuracy with clinical signs such as difficulty looking up, walking, initiating urination and chest tightness. MRI remains the gold standard for diagnosis, offering a sensitivity of 100% and a specificity of 80%. Additionally, specific self-report items like difficulty walking for 15 min and urinary incontinence have significant positive likelihood ratios, indicating a moderate to large increase in the likelihood of cervical myelopathy. However, cervicogenic headaches present a diagnostic challenge due to overlapping symptomatology with other headache types. Symptoms such as unilateral headache, pain triggered by neck movement and reduced cervical range of motion are highly sensitive and can help rule out other conditions. Specificity improves when symptoms include pain over the greater occipital nerve or in the posterior neck region, along with ipsilateral neck, shoulder and arm pain. Combining clinical symptoms and diagnostic criteria, along with imaging or greater occipital nerve blockades, enhances diagnostic accuracy in chronic pain conditions.

Our results revealed that fatty infiltration had the highest discriminatory capacity, with a ROC value of 0.723, indicating a moderate ability to distinguish between the two groups. However, the sensitivity and specificity were not particularly high, suggesting that while fatty infiltration can be a useful marker, it should not be relied upon solely for diagnosis. The other metrics had ROC values below 0.7, indicating poor discriminatory power. These metrics also demonstrated low sensitivity and specificity, further limiting their utility in the clinical diagnosis of chronic neck pain. These findings can be explained by physiological (muscle size may not be associated with chronic pain as proposed in a recent meta-analysis [46]), technical (muscle size was observed to lead in opposite conclusions depending on the imaging method used [56]) and methodological reasons (muscle contouring accuracy seems to be associated with fat infiltration due to loss of clarity in visualizing the fascial layer between the semispinalis cervicis and the deep neck extensors [57]). Therefore, while certain US and SWE features can provide some insight, their overall effectiveness in diagnosing chronic neck pain remains limited. Considering that approximately 80% of the information necessary to identify the source of symptoms is found in the subjective history and the physical examination [55], clinicians should emphasize a strong and structured subjective history paired with a thorough physical examination and use US as a supplement to evaluate the source during the treatment, instead of a tool to identify the source of pain.

### Limitations

Although the sample size of this study was reasonably large, the design is not free from limitations, which should be acknowledged. The most important limitation is that the CM was assessed only at the C4-C5 level, since reliability reports show this level to be the most reliable and comparable to magnetic resonance values [23,51,57,58,59]. Studies that focus on single muscles or levels may not provide a complete picture of muscle morphology, composition and stiffness, limiting its applicability to clinical practice. Another limitation of this study is that a single muscle was assessed. Further research is needed to consider other cervical muscles. In addition, the comparisons made with previous studies should be carefully interpreted, as deep neck extensor muscle morphology has been classically evaluated without distinction between semispinalis capitis, cervical multifidi and short rotators [50]. This lack of differentiation may explain inconsistencies depending on the imaging method used. Finally, the groups analyzed in this study had a significant gender representation difference between both groups. Although this difference had no impact on most of the demographic features between groups, the group with a higher percentage of males (controls) was significantly taller than the group with a higher percentage of females (cases). Since both genders exhibited significant differences for all US and SWE metrics, this imbalance should be considered in the interpretation of the results obtained and future studies should control these differences to ensure non-biased conclusions.

## 5. Conclusions

Although muscle area, perimeter, mean echo intensity, Young’s modulus and SWS metrics were similar between individuals with chronic idiopathic neck pain (cases) and asymptomatic subjects (controls), the neck pain group exhibited significantly higher levels of fatty infiltration. Further, gender differences were noted across nearly all sonographic variables, with males generally having larger muscle areas and perimeters, higher mean echo intensities and more fatty infiltrations than females. Minimal differences were found between the left and right sides of the muscle, with only a slight asymmetry in area. Therefore, the lack of interactions between group, gender and side may indicate that chronic neck pain is more likely related to functional changes rather than structural differences. In terms of the diagnostic accuracy of ultrasound (US) and shear wave elastography (SWE), fat infiltration showed the highest discriminatory ability, with a ROC value of 0.723, indicating a moderate capacity to differentiate between the two groups. However, the sensitivity and specificity were not particularly high, suggesting that while fatty infiltration can be a useful marker, it should not be solely relied upon for diagnosis. The other metrics had ROC values below 0.7, indicating poor discriminatory capability, with low sensitivity and specificity further limiting their usefulness in clinically diagnosing chronic neck pain.

## Figures and Tables

**Table 1:** Participants’ sociodemographic, clinical and sonographic features.

Variables	Cases (*n* = 174)			Controls (*n* = 142)			Between-Group Differences
	Males (*n* = 64)	Females (*n* = 110)	*Difference*	Males (*n* = 91)	Females (*n* = 51)	*Difference*	
*Demographic Data*							
Age (y)	23.2 ± 8.2	23.1 ± 7.0	0.0 (−2.2; 2.4) *p* = 0.957	22.9 ± 5.6	22.0 ± 4.9	0.3 (−1.5; 2.1) *p* = 0.751	0.9 (−0.5; 2.4) *p* = 0.214
Height (m)	1.79 ± 0.06	1.64 ± 0.05	0.1 (0.13; 0.16) *p* < 0.001	1.78 ± 0.06	1.66 ± 0.06	0.13 (0.10; 0.15) *p* < 0.001	0.04 (0.02; 0.06) *p* < 0.001
Weight (kg)	79.4 ± 20.4	60.6 ± 11.3	18.7 (14.0; 23.5) *p* < 0.001	75.3 ± 10.8	61.1 ± 9.5	14.2 (10.6; 17.8) *p* < 0.001	2.7 (0.8; 6.1) *p* = 0.133
BMI (kg/m^2^)	24.7 ± 7.0	22.4 ± 4.2	2.3 (0.6; 4.0) *p* = 0.008	23.5 ± 3.0	22.2 ± 3.4	1.3 (0.2; 2.4) *p* = 0.022	0.3 (−0.7; 1.3) *p* = 0.600
*Clinical Data*							
NDI (0–100)	27.0 ± 16.8	27.4 ± 15.2	0.4 (−4.5; 5.3) *p* = 0.878				
VAS (0–10)	5.1 ± 1.4	5.5 ± 1.6	0.4 (0.0; 1.0) *p* = 0.052				

**Table 2:** Analysis of cervical multifidus sonographic features.

Variables	Cases (*n* = 174)	Controls (*n* = 142)	Between-Group Differences			
			Case/Control	Male/Female	Left/Right	Group*Gender*Side
Area	1.05 ± 0.22	1.08 ± 0.22	$F=0.98;\;p=0.322;\;\eta_{p}^{2}$ = 0.006	$F=63.54;\;p<\;0.001;\;\eta_{p}^{2}$ = 0.295	$F=4.39;\;p=0.038;\;\eta_{p}^{2}$ = 0.028	$F=0.34;\;p=0.557;\;\eta_{p}^{2}$ = 0.002
Perimeter	4.99 ± 0.59	4.99 ± 0.63	$F=1.14;\;p=0.287;\;\eta_{p}^{2}$ = 0.007	$F=40.10;\;p<\;0.001;\;\eta_{p}^{2}$ = 0.209	$F=0.03;\;p=0.848;\;\eta_{p}^{2}$ = 0.000	$F=0.91;\;p=0.340;\;\eta_{p}^{2}$ = 0.006
MEI	52.93 ± 15.5	50.37 ± 14.94	$F=1.90;\;p=0.170;\;\eta_{p}^{2}$ = 0.012	$F=16.49;\;p<\;0.001;\;\eta_{p}^{2}$ = 0.098	$F=1.28;\;p=0.259;\;\eta_{p}^{2}$ = 0.002	$F=0.38;\;p=0.538;\;\eta_{p}^{2}$ = 0.002
Fatty Infiltration	26.83 ± 10.11	20.47 ± 7.41	$F=23.31;\;p<\;0.001;\;\eta_{p}^{2}$ = 0.133	$F=6.01;\;p=0.015;\;\eta_{p}^{2}$ = 0.038	$F=0.96;\;p=0.328;\;\eta_{p}^{2}$ = 0.006	$F=0.09;\;p=0.753;\;\eta_{p}^{2}$ = 0.001
YM	40.74 ± 28.96	33.89 ± 23.21	$F=0.04;\;p=0.839;\;\eta_{p}^{2}$ = 0.000	$F=13.67;\;p<\;0.001;\;\eta_{p}^{2}$ = 0.086	$F=0.01;\;p=0.912;\;\eta_{p}^{2}$ = 0.000	$F=0.06;\;p=0.807;\;\eta_{p}^{2}$ = 0.000
SWS	3.46 ± 1.15	3.12 ± 1.03	$F=0.00;\;p=0.987;\;\eta_{p}^{2}$ = 0.000	$F=14.87;\;p<\;0.001;\;\eta_{p}^{2}$ = 0.092	$F=0.15;\;p=0.699;\;\eta_{p}^{2}$ = 0.000	$F=0.14;\;p=0.708;\;\eta_{p}^{2}$ = 0.001

MEI: mean echo intensity; SWS: shear wave speed; YM: Young’s modulus.

**Table 3:** Discriminant capacity of cervical multifidus analysis with B-mode ultrasound imaging and shear wave elastography to differentiate cases with chronic neck and asymptomatic individuals.

Variables	Area	Perimeter	Mean Echo Intensity	Fat Infiltration	Young’s Modulus	Shear Wave Speed
ROC value	0.475	0.500	0.587	0.723	0.576	0.591
95% CI	0.385–0.565	0.409–0.590	0.498–0.675	0.644–0.803	0.487–0.664	0.503–0.679
Cut-off point	0.77	5.48	49.22	25.77	21.86	2.51
Significance	0.588	0.997	0.048	0.000	0.094	0.044
Youden Index	0.047	0.090	0.210	0.392	0.123	0.185
Sensitivity	0.84	0.24	0.415	0.598	0.750	0.804
Specificity	0.79	0.21	0.205	0.205	0.606	0.620
Positive LR	4.00	0.30	0.52	0.75	1.90	2.11
Negative LR	0.20	3.61	2.85	1.96	0.41	0.31

CI: confidence interval; LR: likelihood ratio; ROC: receiver operating characteristic.

## Data Availability

The raw data supporting the conclusions of this article will be made available by the authors on request.
